# Comparative Safety Profiles of Anti-Amyloid Therapies in Early Alzheimer’s Disease (AD): A Detailed Systematic Review and Meta-Regression Analysis of Amyloid Related Imaging Abnormalities (ARIA) – Incidence and Infusion Reactions for Lecanemab, Donanemab, and Aducanumab

**DOI:** 10.1192/bjo.2025.10235

**Published:** 2025-06-20

**Authors:** Sathyan Soundara Rajan, Sneh Babhulkar, Gaurav Uppal, Sharmi Bhattacharyya, Asha Devi Dhandapani

**Affiliations:** 1 BCUHB, Wrexham, United Kingdom; 2 Greater Glasgow and Clyde NHS Trust, Glasgow, United Kingdom; 3 Satyam Hospital, Ludhiana, India

## Abstract

**Aims:** Alzheimer’s disease continues to be a huge health concern around the globe. Although similar recent developments in anti-amyloid therapy are promising, there is concern about some side effects like ARIA. The purpose of this meta-analysis was to systematically review and compare the safety characteristics of three of the most promising anti-amyloid drugs: lecanemab, donanemab and aducanumab, specifically, the frequency of ARIA-E (oedema) and ARIA-H (haemorrhage).

**Methods:** The present systematic review included only high-quality randomized controlled trials, randomized controlled trials with non-parametric data, and meta-analyses. Data was analysed and visualized by using forest plots, funnel plots and bubble plots. The degree of heterogeneity was examined by I^2^ of statistics.

**Results:** The antibody lecanemab had the lowest ARIA-E rate at 12.6% and the highest infusion reaction rate at 26.4%. Mild ARIA-E was evident in 24.0% of the participants, whereas mild ARIA-H was detected in 19.7% of the subjects.

Of the four candidates, aducanumab showed the highest rate in ARIA-E (30.7%) but the lowest rate in infusion reaction (1.2%). The overall ARIA-H rates appeared to be moderate for all drugs (17.3–19.7%).

The odds ratios in the forest plot were above 1 for all the outcomes suggesting increased adverse event risk. There was moderate to high heterogeneity in all the studies examined (I^2^ = 84.7%).

**Conclusion:** In essence, the risks and benefits of each drug are different. The ARIA-E risk associated with lecanemab is lower than with aducanumab; however, infusion reaction rates remain high. There is reason to believe that such an aggressive ARIA-E profile would ultimately restrict the value of aducanumab and lecanemab. However, donanemab does bring a kind of medium between the two. This review emphasizes the importance of individualized interventions in treating Alzheimer’s dementia.

Future Directions: More research is thus needed to understand some of the factors that have confounded heterogeneity, and to find ways of managing some of the risks associated with ARIA. The current study indicates the importance of long-term safety data along with head-to-head comparisons regarding clinical decision-making.

